# Cardiovascular Magnetic Resonance for the Evaluation of Arrhythmogenic Substrates in Patients with Systemic Autoimmunity: An Update

**DOI:** 10.31083/j.rcm2410290

**Published:** 2023-10-12

**Authors:** George Markousis-Mavrogenis, Petros P. Sfikakis, Anne Hui Sze Kwong, Alessia Pepe, Marco Matucci-Cerinic, George D. Kitas, Sophie I. Mavrogeni

**Affiliations:** ^1^University Research Institute of Maternal and Child Health and Precision Medicine and UNESCO Chair in Adolescent Health Care, Medical School, National and Kapodistrian University of Athens, Aghia Sophia Children’s Hospital, 11527 Athens, Greece; ^2^Joint Rheumatology, Laikon Hospital, National and Kapodistrian University of Athens, 15772 Athens, Greece; ^3^Circle Cardiovascular Imaging, 75001 Paris, France; ^4^Institute of Radiology, Department of Medicine, University of Padova, 35128 Padova, Italy; ^5^Section of Internal Medicine, Department of Experimental and Clinical Medicine, University of Florence, 50134 Florence, Italy; ^6^Unit of Immunology, Rheumatology, Allergy and Rare Diseases (UnIRAR), IRCCS San Raffaele Hospital, 20132 Milan, Italy; ^7^Epidemiology Department, University of Manchester, M5 4BR Manchester, UK; ^8^Onassis Cardiac Surgery Center, 17674 Athens, Greece

**Keywords:** oedema, ischemia, fibrosis, inflammation, cardiovascular disease, autoimmune disease

## Abstract

Patients with systemic autoimmunity due to autoimmune rheumatic diseases (ARDs) or sarcoidosis frequently present with systemic manifestations including cardiac involvement. Cardiac rhythm disturbances and specifically ventricular arrhythmias (VAs) may affect the prognosis of these patients. Cardiovascular magnetic resonance imaging (CMR) is a non-invasive imaging modality that can provide valuable diagnostic and prognostic information in patients with ARDs or systemic autoimmunity in general. In this narrative review, we briefly present the underlying pathophysiologic mechanisms contributing to arrhythmogenicity in patients with systemic autoimmunity. Furthermore, we discuss recent advances underlying the role and value of CMR for use in the detection and risk stratification of arrhythmogenic substrates in patients with systemic autoimmunity and VAs.

## 1. Introduction

Patients with systemic autoimmunity due to autoimmune rheumatic diseases (ARDs) or sarcoidosis (SRC) frequently present with systemic manifestations, including cardiac involvement. Rhythm disorders and specifically ventricular arrhythmias (VAs) are of critical importance for the prognosis of patients with systemic autoimmunity [1]. Although atrial arrhythmias occur more often in this population, VAs are prevalent among patients with SRC, systemic sclerosis (SSc), systemic lupus erythematosus (SLE), and idiopathic inflammatory myopathies (IIM), while arrhythmia-associated mortality is higher amongst patients with SRC and SSc [1]. As such, a basic understanding of the mechanisms of arrhythmogenesis that may occur in the context of systemic autoimmunity is vital for treating physicians managing patients with ARDs. In addition, the detection of arrhythmogenicity with electrocardiographic testing (either standard 12-lead electrocardiogram or 24 h Holter recordings), should prompt the initiation of additional diagnostic steps. In particular, cardiovascular magnetic resonance imaging (CMR) offers distinct advantages specifically in the setting of autoimmunity, and familiarity with its capabilities can reinforce the diagnostic and risk assessment approach to arrhythmogenicity in these patients [2].

We have previously discussed the role of CMR in the evaluation of arrhythmogenic substrates in patients with ARDs in our previous review 5 years ago [2]. However, the rapid progress in the field prompted us to consider an update of our previous work. In this narrative review, we briefly present the underlying pathophysiologic mechanisms contributing to arrhythmogenicity in patients with systemic autoimmunity. Furthermore, we discuss recent advances underlying the role and value of CMR for use in the detection and risk stratification of arrhythmogenic substrates in patients with systemic autoimmunity and VAs.

## 2. Primary Mechanisms of Arrhythmogenicity in Systemic Autoimmunity

A plethora of factors can increase the probability of arrhythmogenicity in patients with systemic autoimmunity. These are not only limited to disease manifestations of the autoimmune condition, but may also be associated with immunomodulatory medication use.

### 2.1 Arrhythmogenic Inflammatory Cardiomyopathy

Arrhythmogenic inflammatory cardiomyopathy (AIC) is an inflammatory condition that can affect the myocardium in patients with systemic autoimmunity and is characterized by the presence of myocardial inflammation, oedema and/or fibrosis. Non-ischemic myocardial scarring may facilitate the development of VAs through re-entry mechanisms. Additionally, inflammatory processes in the myocardium may facilitate the generation of VAs through various mechanisms, including myocardial oedema, arrhythmogenic autoantibodies and inflammatory channelopathies [3]. The clinical presentation of AIC varies from asymptomatic/oligosymptomatic ventricular extrasystolic beats to severe VAs and sudden cardiac death (SCD) [3]. SCD in this context is due to ventricular tachycardia/fibrillation (VT/VF) and may occur in the absence of known structural or functional heart disease.

AIC most often occurs in patients with SSc or SRC [3]. More recently, VT and SCD have also been described in patients with IIM. In these cases, the myocardial histopathology resembles that of skeletal muscle inflammation and is characterized by active myocardial inflammation, localized or diffuse fibrosis, vasculopathy and intimal proliferation/medial sclerosis of blood vessels [4].

### 2.2 Autonomic Dysfunction

The chronic systemic inflammatory processes observed in the setting of systemic autoimmunity may lead to autonomic nervous system dysfunction, including sympathetic overactivation and/or inadequate parasympathetic response. Furthermore, autoantibody-mediated inhibition of potassium channels, L-type calcium channels, $M_{2}$-cholinergic receptors or β_1_-adrenergic receptors may also promote the development of cardiac arrhythmias [1].

### 2.3 Drug-Induced Arrhythmias

The use of corticosteroids, methotrexate or chloroquine has been associated with the occurrence of VAs [1]. However, hydroxychloroquine did not increase the risk of VAs regardless of treatment duration in patients with rheumatoid arthritis (RA), SLE, or Sjögren syndrome (SS) [5].

### 2.4 Epicardial and/or Microvascular Coronary Artery Disease 

Both epicardial and microvascular coronary artery disease may lead to myocardial ischemia with eventual development of oedema/fibrosis in the myocardium of patients with systemic autoimmunity. Epicardial coronary artery disease may lead to ischemia/oedema/fibrosis in the territory, supplied by the involved epicardial coronary artery, while microvascular coronary artery disease may lead to diffuse ischemia/oedema/fibrosis, due to the involvement of the coronary microcirculation [6]. The co-existence of both epicardial and microvascular coronary artery disease is not unusual and carries a worse prognosis [6, 7].

### 2.5 Left and Right Ventricular Dysfunction and Remodeling

Left ventricular ejection fraction (LVEF) at a cut-off value of ≤35% is used to select patients at increased risk of SCD. Reduced LVEF is a serious risk factor predisposing to inducible VT after ST-segment-elevation myocardial infarction. However, LV enlargement with only moderate LV dysfunction also predisposes patients to VAs and SCD [8]. Lastly, right ventricular (RV) dysfunction (right ventricular ejection fraction ≤35%) was an important factor predicting inducible VT in electrophysiologic studies of patients with LVEF ≤40% after acute ST-segment-elevation myocardial infarction treated with primary angioplasty [9].

## 3. Cardiovascular Magnetic Resonance Imaging for the Assessment of Arrhythmogenic Substrates

CMR is the only non-invasive imaging modality that does not employ ionizing radiation and can evaluate cardiovascular function as well as cardiac tissue characterization in a single examination. It can detect and quantify myocardial ischemia, oedema, and/or fibrosis, in parallel with bi-ventricular function and dimension assessment. Specifically in patients with systemic autoimmunity, CMR allows for the early selection of patients at increased risk for VT/VF. As a consequence, a CMR examination could impact the choice of treatment for both cardioprotective and immunomodulatory interventions in these patients, including the optimal selection of candidates for implantable cardioverter defibrillator (ICD) implantation [2].

Despite the various diagnostic strengths of CMR, it cannot directly visualize the presence and type of cardiac leukocyte infiltrates, as in endomyocardial biopsy. However, CMR can provide information superior to endomyocardial biopsy regarding myocardial disease acuity (presence of oedema), local or diffuse ischemia (presence of perfusion defects) or the presence of chronic fibrotic processes (replacement or diffuse fibrosis). In contrast, endomyocardial biopsy is an invasive procedure, which is prone to both sampling and interpretation errors [10]. In the following section we discuss the primary CMR-derived parameters that can influence clinical decision making in patients with either ischemic or non-ischemic heart disease and cardiac rhythm disturbances.

### 3.1 Bi-Ventricular Function

**Fig. 1.**
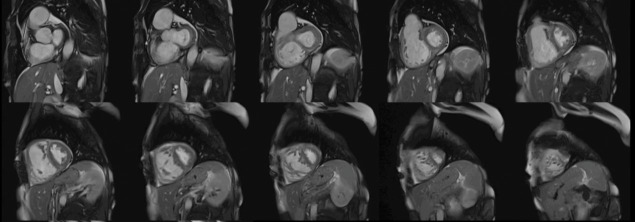
**Biventricular function assessment**. Short axis SSFP for function assessment in a patient with systemic sclerosis and pulmonary hypertension. Dilation of the right ventricle with flattening of the interventricular septum due to pulmonary hypertension can be observed. SSFP, steady-state free precession.

The CMR pulse sequence used for assessment of cardiac function is the balanced steady-state free precession (bSSFP). It represents the gold standard for the evaluation of cardiac anatomy, mass, wall motion, and right/left atrial/ventricular dimensions and function [11]. This is of particular value in patients with autoimmune disease, where RV pathology can play an important role in the generation of VAs and may not be adequately imaged using echocardiography [11] (Fig. 1).

### 3.2 Ischemia Detection

**Fig. 2.**
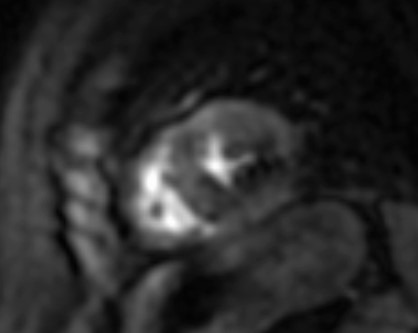
**Stress CMR perfusion**. Adenosine stress perfusion CMR showing a perfusion defect in the inferolateral wall in a patient with systemic sclerosis and ventricular arrhythmias. CMR, cardiovascular magnetic resonance imaging.

CMR can detect myocardial ischemia using either vasodilator perfusion stress testing (adenosine/dypiridamole/regadenoson) or dobutamine stress testing. The most commonly employed test in clinical practice is adenosine stress perfusion, due to its rapid implementation and favorable side effect profile compared with dobutamine. In contrast to other imaging modalities, adenosine stress perfusion CMR has no imaging limitations depending on body dimensions or operator experience. For these reasons, it is the ideal modality for the assessment of both macro- and micro-vascular coronary artery disease, specifically in patients who are unable to exercise, as often occurs amongst patients with systemic autoimmunity [11] (Fig. 2).

Recently, quantitative perfusion CMR has been described and validated against fractional flow reserve (FFR) [12], microspheres [13], and positron emission tomography (PET) [14, 15]. The aim of a quantitative approach is to allow user-independent and reproducible measurements of myocardial perfusion. This is especially important if perfusion abnormalities are diffuse, which precludes reliable visual assessment under normal circumstances. Indeed, a quantitative approach is superior to visual assessment in patients with multi-vessel disease [16]. Similarly, the quantitative approach provided incremental prognostic benefit over a visual approach in an observational study [17]. However, the lack of standardization still remains the main obstacle to more widespread use of quantitative perfusion CMR, particularly regarding the employed acquisition/dosing protocols, the methodology and differences between post-processing analysis software.

On the other hand, PET can provide absolute quantitative myocardial blood flow (MBF) evaluation at rest and during hyperemic vasodilation, with subsequent assessment of myocardial flow reserve (MFR) allowing the non-invasive detection of coronary microvascular disease (CMD). Additionally, the evaluation of hyperemic MBFs and MFR provide a guide for standardized reporting necessary for the diagnosis, treatment, and outcome clinical CMD trials. Lastly, in cases with normal hyperemic MBFs and MFR, further evaluation of the presence of microvascular vasospasm, predominantly with invasive testing, may be considered in the presence of an appropriate clinical scenario [18].

### 3.3 Oedema Detection

**Fig. 3.**
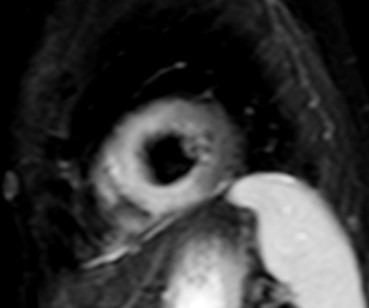
**CMR oedema evaluation using STIRT2.** STIRT2 image with diffuse oedema (interventricular septum, anterior and inferior wall) in a patient with systemic lupus erythematosus myocarditis and ventricular arrhythmias (T2 ratio = 4, normal values <2). STIRT2, short tau inversion recovery; CMR, cardiovascular magnetic resonance imaging.

T2-weighted (T2-W) images are sensitive to the presence of myocardial water and therefore can be used for the assessment of myocardial oedema. The presence of oedema reflects the acute myocardial response to any disease of ischemic, traumatic or inflammatory etiology. Oedema may be diffuse, as in microvascular coronary artery disease, small vessel vasculopathy or myocarditis, localized as in epicardial coronary artery disease (subendocardial/transmural, following the distribution of the involved coronary artery), or subepicardial as in various types of myocarditis [11]. T2-W lesions appear as “bright areas” on short tau inversion recovery (STIRT2) images, where the signal contrast between oedema, normal myocardium and the LV cavity is the best. However, STIRT2 images have limitations, such as a low signal to noise ratio leading to poor contrast between healthy and oedematous areas, susceptibility to magnetic field inhomogeneities and motion artifacts [11] (Fig. 3).

**Fig. 4.**
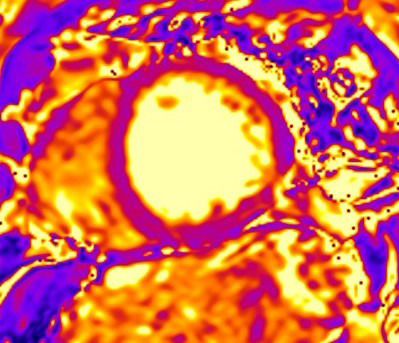
**CMR oedema evaluation using parametric imaging. **T2 mapping in patient with polymyositis and ventricular arrhythmias (T2 mapping = 62 msec, normal values <50 msec). CMR, cardiovascular magnetic resonance imaging.

To overcome these limitations, T2 mapping, a parametric image of each voxel, was developed**. **T2 mapping values are independent of body size and/or heart rate and have good reproducibility [11]; however, they may vary between different scanner types or field strengths and for this reason the definition of individualized normal values for each center is strongly recommended [19, 20]. Increased signal on T2 mapping is an index of myocardial oedema, due to any kind of recent myocardial injury [11] (Fig. 4).

### 3.4 Fibrosis Detection

**Fig. 5.**
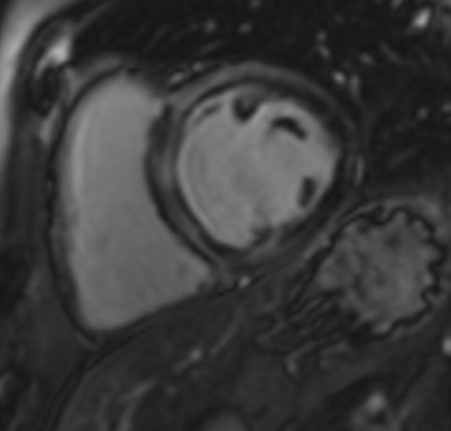
**Replacement fibrosis evaluation using late gadolinium enhancement**. Short axis with extensive late gadolinium enhancement (interventricular septum, anterior wall, inferior wall) in a patient with polymyositis and ventricular arrhythmias.

T1-weighted (T1-W) imaging is used for the anatomical assessment of the heart. Late gadolinium enhanced T1-W images (LGE), taken 8–15 min. After gadolinium-based contrast administration using inversion recovery pulse sequences, permit the detection and quantification of myocardial replacement fibrosis [11] (Fig. 5), if T2-W images in the same regions are negative.

**Fig. 6.**
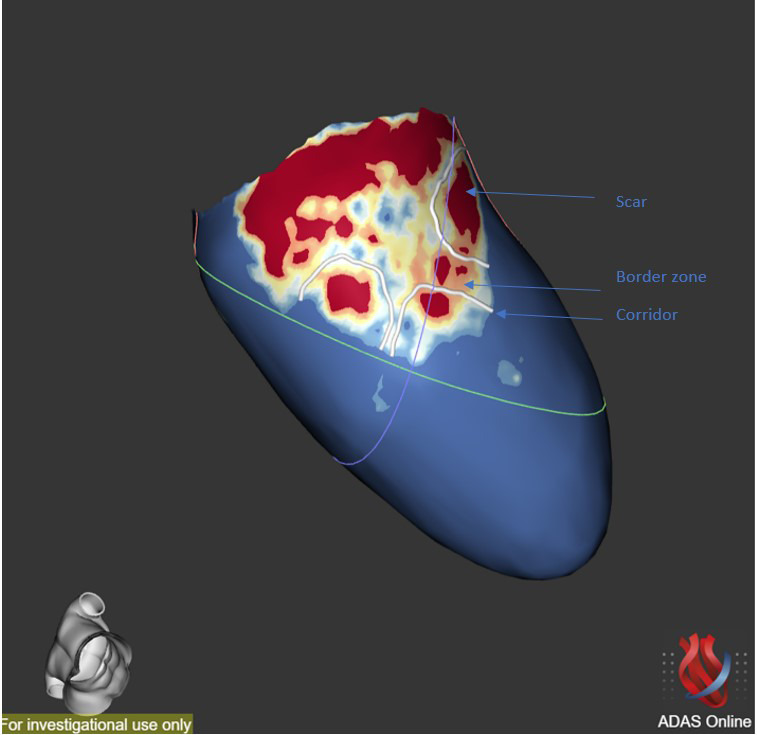
**Scar characterization**. Scar characterization using the ADAS software (v5.11, Galgo Medical, Barcelona, Spain) showing the presence of grey area and scar corridors. Cardiac scar is red, border zone is green-yellow and healthy tissue is blue.

There are several studies presenting scar analysis in patients with VAs post myocardial infarction. A combination of ≥17.2 g border zone mass and the presence of border zone channels was shown to have the best association with VAs in patients with ST-segment elevation myocardial infarction with a 5-time higher prevalence in patients vs. controls. Scar characteristics, including total scar mass, border zone mass and border zone channels were also associated with the development of VAs [21] (Fig. 6). Furthermore, two recent studies [22, 23] have demonstrated that greyzone myocardial fibrosis is strongly associated with VA and SCD. This was also confirmed by other studies showing that scar size/heterogeneity, assessed by LGE, are independent predictors of VAs and CV death, post myocardial infarction [24, 25].

**Fig. 7.**
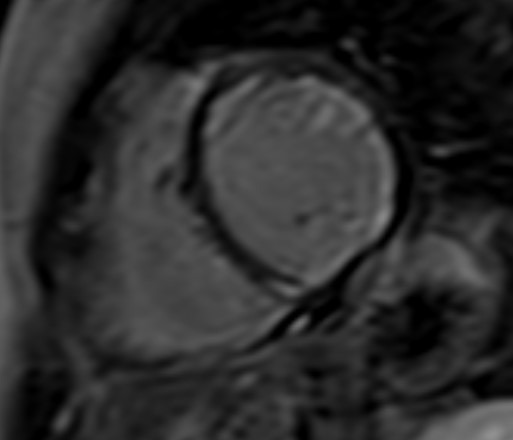
**Subendocardial diffuse fibrosis.** Diffuse subendocardial late gadolinium enhancement in a patient with systemic sclerosis and ventricular arrhythmias.

Although the arrhythmogenic characteristics of LGE have been well studied in patients with myocardial infarction, there is currently limited evidence regarding its characteristics in patients with non-ischemic cardiac disease (NICD), which is common in patients with ARDs. In patients with NICD, the presence of a “ring-like” pattern of LV scar, defined as subepicardial or mid-myocardial LGE involving at least 3 contiguous segments in the same short-axis slice, is associated with idiopathic non-sustained VT [26]. This pattern is not unusual in SSc, SRC and small vessels vasculitides and could potentially explain the increased incidence of VA in these patients [27] (Fig. 7).

Additionally, LGE detects marked expansion of the extracellular space associated with amyloidosis (amyloid deposition and fibrosis) and the LGE pattern can potentially differentiate Amyloid-Transthyretin (ATTR) from AL patients [28], but there are no studies regarding the role of LGE in arrhythmogenesis in these diseases. Lastly, LGE can be detected in pulmonary hypertension, reflecting myocardial disarray with increased collagen content without focal replacement fibrosis at the junction points between the LV and RV, as well as in myocarditis, reflecting inflammation with or without fibrosis [11]. Autopsy studies have revealed that during acute myocarditis, LGE correlates with myocardial necrosis and may co-exist with oedema in T2-W imaging. In chronic myocarditis, LGE correlates with myocardial fibrosis either in the presence or absence of oedema [11].

**Fig. 8.**
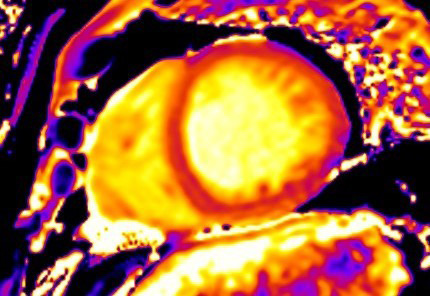
**Detection of microfibrosis using T1 mapping.** T1 mapping in patient with polymyositis and ventricular arrhythmias (T1 map = 1400 ms, normal values <1250 ms).

Although LGE is well-established as the technique of choice for the assessment of replacement fibrosis, it cannot evaluate diffuse fibrosis, because it is based on signal intensity differences between scarred and normal myocardium [11]. To overcome this limitation, parametric imaging was developed including T1 mapping (Fig. 8) and extracellular volume fraction (ECV) quantification [11]. T1 mapping (native/pre-contrast and post-contrast after administration of gadolinium-based contrast agent) provides a quantitative assessment of tissue T1 values and enables identification of diffuse myocardial fibrosis [11]. ECV is calculated using native T1 mapping, post-contrast T1 mapping and the patient’s haematocrit, with the latter preferably having been measured on the same day as the CMR study. ECV is calculated based on the following formula:

$$\begin{array}{c} ECV=\left(1-Hematocrit\right)\times \\ \frac{\left(1/T\,1_{\left(\text{myo post–contrast }\right)}\right)-\left(1/T\,1_{\left(\text{myo pre–contrast }\right)}\right)}{\left(1/T\,1_{\left(\text{blood post–contrast }\right)}-1/T\,1_{\left(\text{blood pre–contrast }\right)}\right)} \end{array}$$

Apart from amyloidosis, elevated ECV values can be due to excessive collagen deposition as in diffuse fibrosis observed mainly in patients with SSc, but also other autoimmune diseases [11].

Lastly, in patients with NICD without LGE, diffuse fibrosis, estimated using post-contrast T1 mapping, correlates with voltage abnormalities identified at electroanatomic mapping and can affect the post-ablation prognosis [29].

### 3.5 Iron Deposition within Chronic Myocardial Infarction (CMI)

Chronic myocardial infarction (CMI) is not unusual in patients with systemic autoimmunity, due to increased atherosclerosis caused by chronic inflammation. Iron deposition within CMI can influence the electric characteristics of the heart. Hypointense cores within CMI, as imaged using balanced steady-state free precession sequences, can be used as a marker of iron deposition and can augment the identification of patients at risk of malignant VAs [30]. However, a hypointense signal within CMI on balanced steady-state free precession sequences could also be due to a black boundary (India ink) artifact indicating fat metaplasia [31]. Thus, it is recommended to confirm the presence of iron using a T2* sequence [32]. Although only preliminary results are currently available, it has been argued that microvasculopathy-related iron deposition in tissues, for example in patients with SSc, may act as a pathogenetic link between microvasculopathy and fibrosis [33].

## 4. Can CMR Identify the Arrhythmogenic Substrate of Ventricular Tachycardia in Patients with Systemic Autoimmunity?

Although numerous publications have addressed and demonstrated the clinical value of CMR for the detection of cardiovascular disease in patients with systemic autoimmunity, scarce evidence exists as to its role in the assessment of arrhythmogenicity in these patients. Notably, evidence from studies with adequate electrocardiographic monitoring with concomitant use of CMR is severely lacking. The majority of published data mainly describe the underlying abnormalities detected by CMR in the presence of VA in patients with SRC and systemic sclerosis (SSc).

In a study by our group, a population of 80 consecutive patients with non-sustained ventricular tachycardia (NSVT) and preserved LVEF, 40 with various ARDs and 40 with non-ARD-related cardiac diseases, CMR-based myocardial scar characterization identified a non-ischemic and ischemic LGE pattern as the most predominant fibrotic pattern in the former and latter, respectively. Patients with ARDs had significantly higher native T1 mapping and ECV, independent of various confounding factors [27].

The clinical value of LGE has particularly been demonstrated in patients with SRC. Recently the quantification of LGE as a percentage of LV mass has been incorporated as an adjunct parameter in clinical practice guidelines for ICD implantation in these patients [34]. Furthermore, LGE in the RV was an independent predictor of appropriate shock therapy [35]. Additionally, the presence of LGE is associated with an increase in both all-cause mortality and arrhythmogenicity in these patients [36, 37], although others only reported an association of LGE in the RV free wall with VT occurrence [38]. Lastly, in a cohort of 290 patients with known or suspected SRC, in those with LVEF >35%, an LGE value >5.7% of LV mass provided the highest discriminating performance for the composite end point [34].

Regarding SSc, the Scleroderma Arrhythmia Clinical Utility Study (SAnCtUS), a prospective multicenter study that included 150 consecutive patients with SSc from eight European centers, demonstrated that T2 ratio and %LGE had the greatest utility as independent predictors of rhythm disturbances [39]. Another study of 32 patients with SSc without overt cardiac disease did not identify definite associations between focal or diffuse myocardial fibrosis and arrhythmias, although the study may have been underpowered considering that only 7 patients experienced VAs [40].

There are very sparse reported findings regarding the relationship between VAs and CMR findings in patients with vasculitides. In a study of 20 patients with eosinophilic granulomatosis with polyangiitis in remission, 90% showed some form of cardiac involvement when examined with CMR. LVEF was lower on average compared with controls, LGE in the LV was detected in 89% of patients, and some also showed signs of ongoing inflammation (increased early gadolinium enhancement) and edema (T2-weighted imaging). Holter recordings revealed both supraventricular and ventricular arrhythmias [41].

## 5. Conclusions

Cardiac arrhythmogenesis is characterized by various substrates in patients with systemic autoimmunity due either to ARDs or SRC. These may vary, depending on the ischemic or non-ischemic background and the presence of acute, chronic and/or concomitant acute/chronic cardiac disease. CMR is the only non-invasive imaging modality that can evaluate these arrhythmogenic substrates and thus offers incremental diagnostic and prognostic value to the clinician. Despite these capabilities, clinical research in patients with systemic autoimmunity is very sparse and much still remains to be elucidated. In addition, CMR may be used for the identification of high-risk patients that could benefit from ICD implantation, but with the exception of SRC, no incorporation of CMR findings into practice algorithms has, as of yet, occurred. Thus, to conclude, the role of CMR in the evaluation of arrhythmogenicity in patients with systemic autoimmunity is very promising but thus far greatly underdeveloped, and concerted scientific efforts are required in order to distil potential clinical benefits from the application of CMR in clinical practice.
